# ﻿Protist ecology in Patagonian peatlands: pH, organic phosphorus, and sulfate as key drivers of testate amoeba diversity in undisturbed ecosystems

**DOI:** 10.3897/zookeys.1239.146538

**Published:** 2025-05-21

**Authors:** Leonardo D. Fernández, Erwin Domínguez, Antonio Parra-Gómez, Enrique Lara

**Affiliations:** 1 Núcleo de Investigación en Sustentabilidad Agroambiental (NISUA), Facultad de Medicina Veterinaria y Agronomía, Universidad de Las Américas, Manuel Montt 948, Providencia, Santiago, Chile Universidad de Las Américas Santiago Chile; 2 Instituto de Investigaciones Agropecuarias INIA - Kampenaike, Punta Arenas, Chile Instituto de Investigaciones Agropecuarias INIA - Kampenaike Punta Arenas Chile; 3 Facultad de Ciencias, Universidad Austral de Chile, Av. Rector Eduardo Morales Miranda 23, Valdivia, Chile Universidad Austral de Chile Valdivia Chile; 4 Real Jardín Botánico (RJB-CSIC), Madrid, Spain Real Jardín Botánico (RJB-CSIC) Madrid Spain

**Keywords:** Biodiversity patterns, Chilean Patagonia, ecological gradients, environmental drivers, microbial communities, peatlands, *Sphagnum*-dominated peatlands

## Abstract

Peatlands in southern Chile, particularly in the remote Aysén region, are unique ecosystems that remain understudied despite their ecological significance as natural laboratories. These near-pristine environments serve as essential water reservoirs and harbor largely unexplored microbial diversity. Testate amoebae, a group of shelled protists, play key roles in nutrient cycling and are highly sensitive to environmental changes, making them valuable bioindicators. However, the ecological drivers shaping their diversity and community composition in Chilean peatlands remain poorly understood. This study investigated the spatial distribution and diversity of testate amoebae across five peatlands in the Aysén region (44°S and 49°S; Southern Patagonia, Chile). We recorded 73 morphospecies, including typical southern taxa such as *Alocoderacockayni*, *Apoderavas*, *Argynniagertrudeana*, *Certesellacertesi*, and *C.martiali*. Redundancy analysis revealed that organic phosphorus, pH, and sulfate are the most influential factors shaping testate amoeba communities. Beta diversity analysis indicated significant spatial turnover among sites, suggesting that local environmental gradients strongly influence species distribution. Interestingly, *Sphagnum*-dominated peatlands exhibited higher diversity compared to those with mixed vegetation (vascular plants and bryophytes). Our findings fill a critical gap in understanding microbial biodiversity in Chilean peatlands and highlight the importance of conserving these ecosystems as reservoirs of microbial diversity and natural archives of environmental change.

## ﻿Introduction

Temperate peatlands, particularly those dominated by *Sphagnum* mosses, are wetlands characterized by the accumulation of organic matter, primarily from partially decomposed plants, under waterlogged and anaerobic conditions (Charman 2002; Page et al. 2011). These unique ecosystems are primarily found in boreal and temperate regions (Xu et al. 2018), although peatlands in general are globally distributed, including extensive tropical peatlands (Swindles et al. 2016). Despite their global distribution, peatlands occupy only about 3% of the Earth’s terrestrial surface but account for nearly 30% of the world’s soil carbon (Mitra et al. 2005; Xu et al. 2018). This disproportionate carbon storage underscores their critical role in the global carbon cycle, acting as both carbon sinks and sources under changing environmental conditions (Mitra et al. 2005; Page et al. 2011).

The ecological importance of peatlands extends beyond carbon sequestration and storage. They provide essential ecosystem services, including water regulation, habitat provision for specialized flora and fauna, and biodiversity hotspots (Bonn et al. 2016; Grzybowski and Glińska-Lewczuk 2020; Reid and Torres 2021). Peatlands are home to numerous species adapted to the nutrient-poor and acidic conditions of these environments, many of which are rare or endemic (Parish et al. 2008; Singer et al. 2018; León et al. 2021). Furthermore, they act as natural archives, preserving pollen, spores, and other biotic materials that offer insights into historical climate and vegetation patterns (Charman 2002; Swindles et al. 2016; McCulloch and Reid 2021).

However, peatlands are increasingly threatened by anthropogenic activities. Drainage for agriculture and forestry, peat extraction for fuel and horticulture, and infrastructure development have led to widespread degradation (UNEP 2024). Climate change poses an additional threat, as rising temperatures and altered precipitation patterns exacerbate peatland drying and carbon release (Parish et al. 2008). Globally, it is estimated that more than 15% of peatlands have been degraded, contributing significantly to greenhouse gas emissions and biodiversity loss (UNEP 2024).

In South America, peatlands are primarily concentrated in the temperate southern regions, particularly in Chile (León et al. 2021). Chile harbors extensive peatland systems, covering approximately 10,470 km^2^ (León et al. 2021). Found mainly in remote and inaccessible regions, these ecosystems are prominent in the Aysén region of southern Chile, between 44°S and 49°S (Villarroel et al. 2021; Mansilla et al. 2023). The relative inaccessibility of these peatlands has preserved their natural conditions, with minimal human disturbance (León et al. 2021). Aysén’s peatlands, dominated by *Sphagnum* mosses, represent a significant portion of Chile’s peatland coverage (Domínguez and Martínez 2021) and are considered a key landscape feature to be conserved due to their ecological and carbon storage functions (Hoyos-Santillán et al. 2019; Hoyos-Santillán and Mansilla 2021).

Despite their ecological importance, the biodiversity of Chilean peatlands remains poorly documented (Domínguez and Martínez 2021; León et al. 2021; Mansilla et al. 2023). This knowledge gap is particularly evident in Aysén’s peatlands, where biodiversity assessments have largely focused on macroscopic organisms such as animals and plants (Domínguez and Vega 2015; Domínguez and Silva 2021; Larraín and Vargas 2021; Ortiz and Domínguez 2021; Raimilla 2021), whereas unicellular eukaryotes (protists) have received comparatively limited attention (Campello-Nunes et al. 2021, 2022; Fernández 2021). Among the latter, testate amoebae are one of the most studied protist groups globally, yet they remain largely underexplored in Chilean ecosystems, including its peatlands (but see Fernández et al. 2015).

Testate amoebae are a polyphyletic group of shelled protists that inhabit a variety of terrestrial and aquatic environments, playing essential roles in nutrient cycling, organic matter decomposition, and microbial food webs (Meisterfeld 2002a, 2002b). Due to their sensitivity to environmental changes, they are widely recognized as excellent bioindicators for monitoring ecosystem health, with applications in both the Northern and Southern Hemispheres (Turner and Swindles 2012; Valentine et al. 2013; Van Bellen et al. 2014; Bamforth 2015; Zheng et al. 2019; González-Miguéns et al. 2024; McKeown et al. 2024). Additionally, their well-preserved shells in peatlands and sediments provide valuable records for paleoenvironmental studies, which have been used to infer past climate variations and ecosystem dynamics in both hemispheres (Mitchell et al. 2008; Van Bellen et al. 2014; Charqueño-Celis et al. 2022; Kuuri-Riutta et al. 2022; Tsyganov et al. 2022; Charqueño-Celis et al. 2024).

In South America, studies have established their potential as bioindicators for past hydrological conditions in peat bogs (Van Bellen et al. 2014) and for assessing hydrothermal activity in Argentina (Sagasti et al. 2024). Similarly, testate amoebae have been used to evaluate the ecological impact of volcanic activity in Chile and Argentina (Delaine et al. 2016; Charqueño-Celis et al. 2022) and their environmental responses have been studied in paleolimnological reconstructions in Patagonia (Charqueño-Celis et al. 2024). Their bioindicator potential has also been demonstrated in the temperate rainforests of New Zealand and Tasmania (Bamforth 2015) and in southeastern Australia, where they have been used as proxies for reconstructing past water-table fluctuations (Zheng et al. 2019). Additionally, testate amoebae have been recognized as promising bioindicators in New Zealand peatlands, where they have been studied alongside vascular plants for biomonitoring applications (McKeown et al. 2024).

Previous studies on Chilean testate amoebae have largely concentrated on their taxonomy and diversity (Certes 1889; Izquierdo 1906; Wailes 1913; Jung 1942; Hoogenraad and de Groot 1951; Bonnet 1966; Zapata and Rudolph 1986; Zapata and Matamala 1987; Zapata and Crespo 1990; Golemansky and Todorov 1996; Zapata et al. 2002; Zapata 2005; Zapata et al. 2007a, 2007b; Zapata and Fernández 2008; Zapata et al. 2008; De Smet and Gibson 2009; Santibañez et al. 2011; Fernández et al. 2012; Chatelain et al. 2013; Fernández 2015; Fernández 2021). However, research investigating the ecological drivers of their diversity and community composition remains scarce at both local and regional scales (Fernández et al. 2016). In Chile, research investigating the ecological drivers shaping testate amoeba communities in peatlands is notably scarce, with only one study published to date (Fernández and Zapata 2011), leaving a critical gap in our understanding of these ecosystems.

This study aims to address this gap by investigating the diversity and community composition of testate amoebae in the peatlands of the Aysén region, Chile. Specifically, we seek to identify the environmental drivers influencing their spatial distribution and diversity patterns. By focusing on a region with minimal human impact, this research provides a unique opportunity to study the natural dynamics of testate amoeba communities in undisturbed peatlands. Our findings will contribute to the broader understanding of protist ecology for peatland ecosystems in Chile and beyond. Unlike studies that emphasize microtopographical variation and hydrological gradients, our approach is based on composite sample collection, allowing us to assess the influence of other environmental factors, such as organic phosphorus and sulfate, which have been less explored in testate amoeba research.

## ﻿Materials and methods

### ﻿Study sites

**Figure 1.**
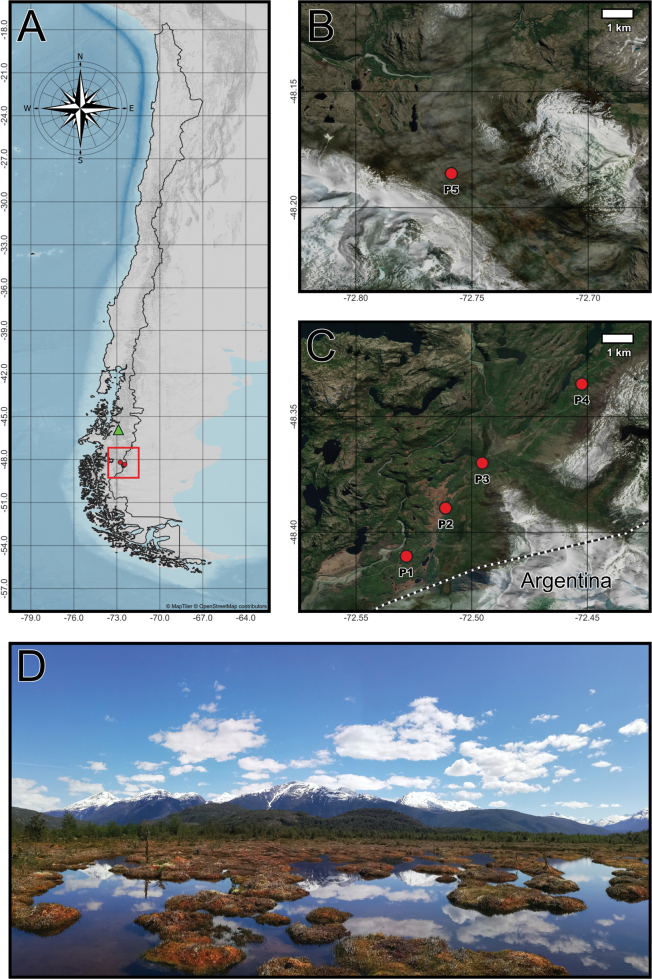
Spatial distribution of study sites in the peatlands of the Aysén Region, Chile: **A** regional map showing the location of the studied peatlands (red dots) within southern Chile, highlighted by the red box. The green triangle marks the location of Hudson Volcano, the most active volcano in the region; **B** satellite image focusing on the location of site P5 in the southern section of the study area; **C** satellite image showing the distribution of study sites P1–P4 in proximity to the Chile-Argentina border. Dotted lines indicate the international boundary; **D** panoramic photograph of a representative peatland ecosystem within the study area, illustrating the landscape’s characteristic vegetation and hydrological features. The map was constructed in QGIS 3.8.21 by combining multiple layers from the MapTiler© OpenStreetMap contributors©.

The study was conducted in five peatlands (P1, P2, P3, P4, P5) located in the remote Aysén region of southern Chile, South America during November 2023 (Fig. 1). These peatlands are natural ecosystems situated in areas of difficult access and show no evidence of human exploitation or modification, thus preserving their near-pristine conditions (Domínguez and Martínez 2021). P1–P4 are peatlands dominated by *Sphagnum* mosses, while P5 represents a mixed peatland characterized by a combination of *Sphagnum* and vascular plants (Domínguez and Martínez 2021). All five peatlands are ombrotrophic, receiving nutrients primarily from the atmospheric deposition rather than groundwater inputs.

These peatlands exhibit diverse plant communities (Domínguez and Silva 2021). The vegetation includes species such as *Sphagnummagellanicum*, *Empetrumrubrum*, *Gaultheriaantarctica*, *Carexmagellanica*, *Tetronciummagellanicum*, and the lichen *Cladoniapycnoclada*. Notably, the orchid *Chloraeagaudichaudii* stands out as an endemic species, while *Carexcanescens* is the only introduced species recorded. Several species in the peatlands are of conservation concern, classified as vulnerable (Domínguez and Silva 2021). These include the ferns *Hymenophyllumsecundum* and *Schizaeafistulosa*, as well as the conifers *Lepidothamnusfonkii* and *Pilgerodendronuviferum*.

### ﻿Field methods

At each site, a 10 m × 10 m plot was established in representative areas of the peatland, selected based on environmental characteristics such as hydrology, vegetation, and microhabitats. Within each plot, both water and testate amoeba samples were collected to ensure consistency in environmental assessments.

For water sampling, triplicate water samples were collected within the plot using acid-washed polyethylene bottles to minimize contamination. Parameters such as pH and temperature were measured in situ using a portable multiparameter probe.

For testate amoebae, 10 random surface-layer samples (3–5 cm depth) of *Sphagnum* moss or dominant bryophytes were collected within the plot and combined into a single composite sample per site. These samples were stored in sealed plastic bags, kept out of direct sunlight, and maintained at 2–4 °C. The bags containing the samples were periodically opened to allow air exchange and prevent anaerobic conditions that could adversely affect testate amoeba survival.

### ﻿Laboratory methods

In the laboratory, water samples were processed according to standard environmental monitoring protocols. Samples for dissolved ions (e.g., dissolved silica, alkalinity, sodium, potassium, magnesium, calcium, fluoride, chloride, and sulfate) and nutrient concentrations (e.g., ammonium, nitrate-nitrite nitrogen, organic nitrogen, orthophosphate phosphorus, organic phosphorus) were filtered through 0.45 µm membranes and preserved at 4 °C or acidified (using H_2_SO_4_ or HCl) depending on the target analyte. Dissolved organic carbon was measured using UV-Vis spectrophotometry and total organic carbon analysis. All water samples were analyzed in a certified laboratory using ion chromatography, spectrophotometry, and ICP-OES (Inductively Coupled Plasma Optical Emission Spectrometry). Detailed values of these environmental variables are presented in Suppl. material 1: table S1.

Testate amoeba samples were processed by adding distilled water, shaking, and washing over a 500 µm mesh sieve to remove debris and large particles. The sieved material was mounted on microscope slides and examined under a light microscope. Only live individuals (i.e., active individuals or shells containing cytoplasm) were counted. To estimate absolute abundances, Lycopodium spores were added to the samples as an exotic marker to enable the calculation of testate amoeba concentrations per gram of dry moss, following standard procedures. Species were identified using standard taxonomic keys (Certes 1889; Jung 1942; Mazei and Tsyganov 2006; Zapata et al. 2007a, 2007b; Kosakyan et al. 2020). The resulting abundance data were compiled into a matrix (Suppl. material 1: table S2) for subsequent statistical analyses.

### ﻿Statistical analyses

To evaluate the adequacy of the sampling effort and the proportion of species detected, rarefaction-extrapolation curves were generated using the iNEXT package (Hsieh et al. 2016), with 1000 permutations. This approach produced seamless rarefaction and extrapolation sampling curves of Hill numbers for *q* = 0, assessing the impact of sampling artifacts and the completeness of species detection.

Alpha diversity metrics, including Species Richness, Shannon Diversity Index, and Simpson Diversity Index, were calculated for each site. Species richness reflects the total number of taxa present; the Shannon Index quantifies diversity by incorporating both species richness and evenness; and the Simpson Index measures dominance by estimating the probability that two randomly selected individuals belong to the same species. Species richness was determined by counting the total number of species observed per site using the specnumber function from the vegan package (Oksanen et al. 2022), while Shannon and Simpson indices were computed using the diversity function from the same library. These indices account for both species richness and evenness in community composition. The resulting diversity metrics were visualized as a barplot using the ggplot2 package (Wickham 2016). Data were organized into a long format with the pivot_longer function from the tidyr package (Wickham and Girlich 2023), and a barplot was created using the geom_bar function with metrics on the y-axis and site labels on the x-axis. Each bar represents the calculated diversity value for a given metric and site. The figure serves to visually compare the diversity patterns across the five peatlands (P1–P5). The input data consisted of a species abundance matrix with sites as rows and species as columns.

Beta diversity, i.e., the spatial variation in species composition across peatlands, was investigated using complementary multivariate analyses. To visualize patterns of community composition, a non-metric multidimensional scaling (nMDS) was performed using a Bray–Curtis dissimilarity matrix. The Bray–Curtis matrix was calculated with the vegdist function from the vegan package (Oksanen et al. 2022). The nMDS ordination was generated with the metaMDS function, which optimizes the arrangement of sites in a low-dimensional space based on their dissimilarity in species composition. Colors in the nMDS plot were assigned using k-means clustering (kmeans function), which partitions sites into clusters according to community composition. The final nMDS plot, created with the ggplot2 package (Wickham 2016), provided a clear visualization of spatial patterns in community structure.

To explicitly quantify beta diversity and its underlying components, we employed the betapart package to calculate total beta diversity (β_SOR_) and its additive components: spatial turnover (β_SIM_) and nestedness-resultant dissimilarity (β_SNE_) (Baselga and Orme 2012). The β_SOR_ metric was derived from the Sørensen dissimilarity index, which accounts for both species turnover and nestedness across sites. The spatial turnover component (β_SIM_) was calculated using Simpson’s dissimilarity index, isolating the variation in species composition exclusively driven by turnover. The nestedness component (β_SNE_) was obtained as the difference between β_SOR_ and β_SIM_, capturing the portion of beta diversity attributable to species gain or loss between sites.

To visualize these beta diversity components, bar plots were generated using the ggplot2 package (Wickham 2016). The data were prepared in a long format using the pivot_longer function from the tidyr package (Wickham and Girlich 2023), and the barplots were created with the ggplot function, providing a comprehensive view of the relative contributions of spatial turnover and nestedness to overall beta diversity.

A redundancy analysis (RDA) was conducted to examine the relationship between testate amoebae community structure and environmental variables. Prior to the RDA, multicollinearity among environmental variables was evaluated by calculating pairwise Pearson correlations with the cor function and visualizing results as a heatmap using the corrplot function from the corrplot package (Wei and Simko 2021). Variables with high correlations (*r* > 0.8) were excluded using the select function from the dplyr package (Wickham et al. 2023), retaining temperature, pH, dissolved organic carbon, organic phosphorus, and sulfate for further analysis (Suppl. material 1: fig. S1a). The retained subset was assessed for multicollinearity using the cor function and visualized using corrplot, confirming the absence of high correlations (Suppl. material 1: fig. S1b). A detrended correspondence analysis (DCA) was then performed using the decorana function from the vegan package (Oksanen et al. 2022), revealing a gradient length of 1.304 on the first axis, which justified the use of RDA. The species abundance matrix was Hellinger-transformed with the decostand function (method = “hellinger”) from vegan, while environmental variables were log-transformed using log (x+1) and standardized to zero mean and unit variance using the mutate and scale functions from dplyr (Wickham et al. 2023) and base R, respectively. The RDA was performed with the rda function in vegan, with the Hellinger-transformed abundance matrix as the response variable and the transformed and standardized environmental variables as predictors. The significance of the constrained model, individual axes, and explanatory variables was tested using a one-way analysis of variance (ANOVA; 1,000 permutations; *P* < 0.05) with the anova function in vegan (Oksanen et al. 2022). However, due to the limited number of sampling sites (n = 5), the test could not generate reliable F or *P*-values, as there were insufficient residual degrees of freedom. Given this limitation, we opted not to report the ANOVA results in the manuscript, as any interpretation of significance would be unreliable. All statistical analyses were performed using R v.4.3.1. (R Core Team 2023).

## ﻿Results

### ﻿Sampling artifact analysis

**Figure 2.**
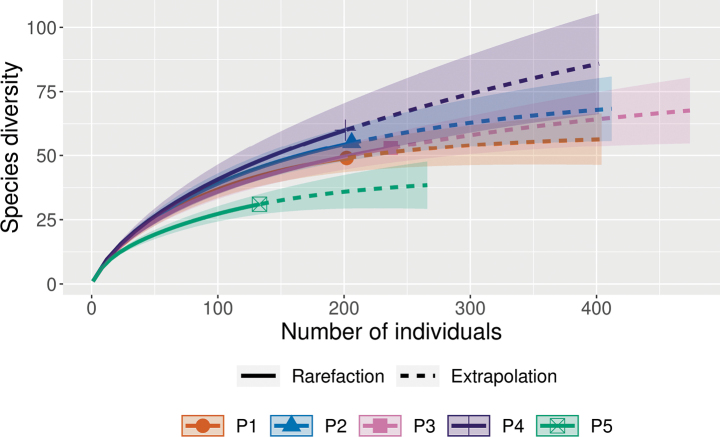
Rarefaction and extrapolation curves for species diversity at peatlands coded as P1–P5. Rarefaction (solid lines) and extrapolation (dashed lines) curves of Hill numbers (q = 0) were generated using the iNEXT package in R, based on species abundance data. Shaded areas represent 95% confidence intervals calculated from 1,000 permutations. The x-axis shows the number of individuals sampled, while the y-axis represents species diversity. Symbols indicate observed species diversity at each site, highlighting sampling completeness and the potential impact of sampling artifacts.

Rarefaction and extrapolation curves generated revealed that the sampling effort was effective in capturing the majority of species diversity across peatlands (Fig. 2). The rarefaction curves indicated that species diversity reached clear asymptotes at sampling sites, reflecting comprehensive sampling coverage. While the extrapolation curves extended beyond observed values, the narrow confidence intervals for most sites underscored the robustness of the data and the reliability of species diversity estimates. These findings highlight the efficacy of the sampling strategy in accurately characterizing local species richness and community structure in peatlands, providing a solid foundation for subsequent analyses.

### ﻿Alpha diversity

**Figure 3.**
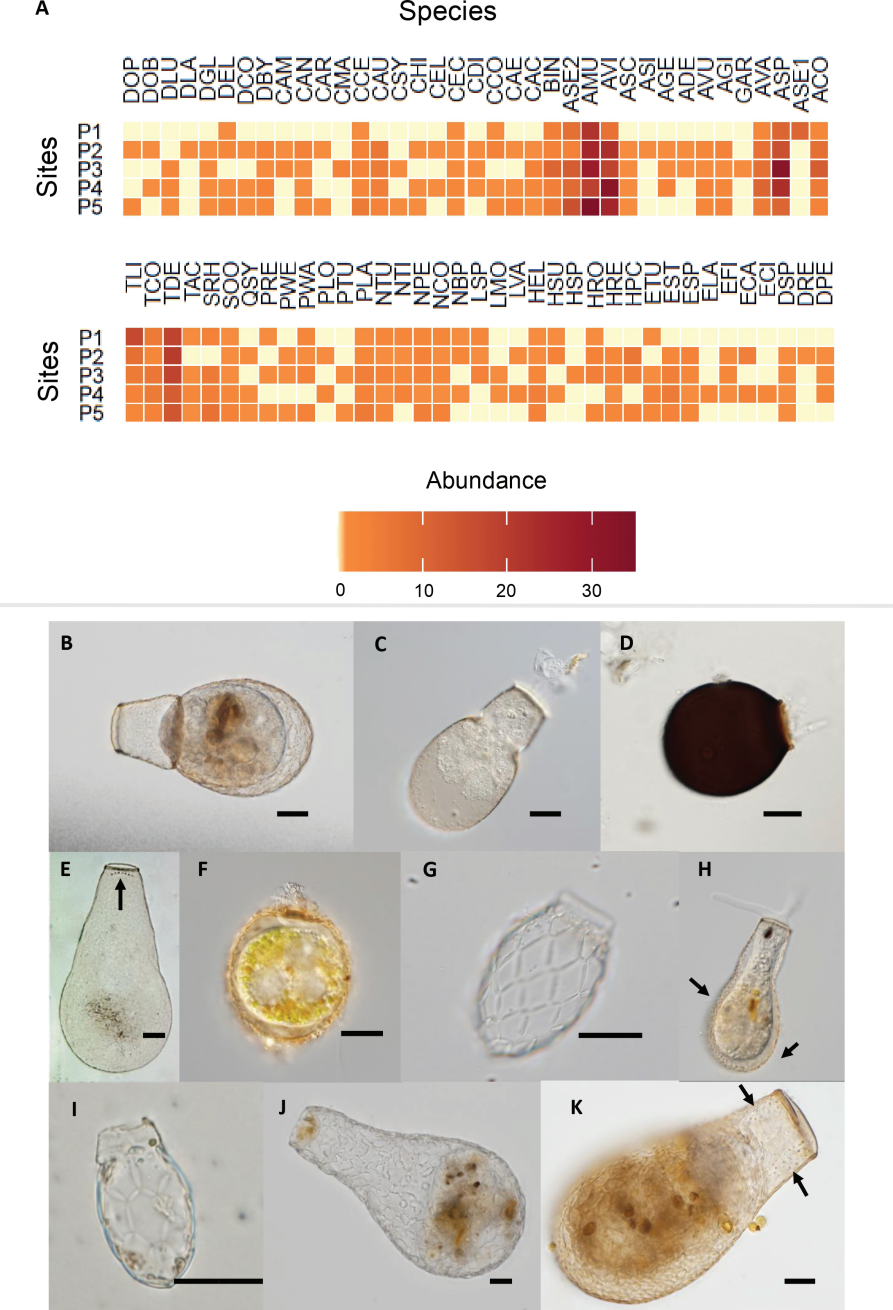
Testate amoeba species: **A** heatmap of species abundance across sites P1–P5, with species names coded for brevity. Species codes correspond to the following names: ACO: *Alocoderacockayni*, ADE: *Argynniadentistoma*, AGE: *Argynniagertrudeana*, AGI: *Arcellagibbosa*, AMU: *Assulinamuscorum*, ASC: *Argynniaschwabei*, ASE1: *Amphitremastenostoma*, ASE2: *Argynniaseminulum*, ASP: *Amphitrema* sp., AVA: *Apoderavas*, AVI: *Argynniavitraea*, AVU: *Arcellavulgaris*, BIN: *Bullinulariaindica*, CAC: *Centropyxisaculeata*, CAE: *Centropyxisaerophila*, CAM: *Cyphoderiaampulla*, CAN: *Cyclopyxisarenata*, CAR: *Cyclopyxisarcelloides*, CAU: *Certesellaaustralis*, CCE: *Certesellacertesi*, CCO: *Centropyxisconstricta*, CEC: *Centropyxisecornis*, CEL: *Centropyxiselongata*, CHI: *Centropyxishirsuta*, CMA: *Certesellamartiali*, CSY: *Centropyxissylvatica*, DBY: *Difflugiabryophila*, DCO: *Difflugiacorona*, DEL: *Difflugiaelegans*, DGL: *Difflugiaglobulosa*, DLA: *Difflugialacustris*, DLU: *Difflugialucida*, DOB: Difflugiacf.oblonga, DOP: *Difflugiaopulenta*, DPE: *Difflugiapenardi*, DRE: *Difflugiaregularis*, DSP: *Difflugia* sp., ECA: *Euglyphacristataacicularis*, ECI: *Euglyphaciliata*, EFI: *Euglyphafilifera*, ELA: *Euglyphalaevis*, ESP: *Euglypha* sp., EST: *Euglyphastrigosa*, ETU: *Euglyphatuberculata*, GAR: *Galeriporaarenaria*, HEL: *Hyalospheniaelegans*, HPC: Heleoperacf.petricola, HRE: *Heleoperarectangularis*, HRO: Heleoperacf.rosea, HSP: *Heleopera* sp., HSU: *Hyalospheniasubflava*, LMO: Lesquereusiacf.modesta, LSP: *Lesquereusiaspiralis*, LVA: *Lagenodifflugiavas*, NBP: *Nebelabarbatapsilonata*, NCO: Nebelacf.collaris, NPE: *Nebelapenardiana*, NTI: Nebelacf.tincta, NTU: *Nebelatubulosa*, PLA: *Padaungiellalageniformis*, PLO: *Padaungiellalongitubulata*, PRE: *Pareuglyphareticulata*, PTU: *Padaungiellatubulata*, PWA: *Padaungiellawailesi*, PWE: *Padaungiellawetekampi*, QSY: Quadrulellacf.symmetrica, SOO: *Sphenoderiaovoidea*, SRH: *Sphenoderiarhombophora*, TAC: *Tracheleuglyphaacolla*, TCO: *Trinemacomplanatum*, TDE: *Tracheleuglyphadentata*, TLI: *Trinemalineare*. Color gradients in the heatmap indicate abundance, from low (light yellow) to high (dark red); **B–K** Micrographs showing morphological details of selected species. The names of the species shown in each panel are as follows: **B***Apoderavas***C***Alocoderacockayni***D***Assulinamuscorum***E***Certesellaaustralis* (note the semicircular row of internal teeth near the lip of the pseudostoma) **F***Amphitremastenostoma***G***Sphenoderiarhombophora***H***Nebelabarbatapsilonata* (note the spines present on the shell) **I***Sphenoderiaovoidea***J***Argynniagertrudeana*, and K. *Certesellamartiali* (Note the rows of teeth on the inside of the shell). Scale bars: 20 µm.

A total of 1,325 individuals were recorded, encompassing 33 genera and 73 species (Fig. 3A; Suppl. material 1: table S1). The genus *Difflugia* was identified as the most diverse in the dataset, comprising 11 recorded species, followed by *Centropyxis*, which included eight species. In contrast, the least diverse genera, represented by only a single species each, were *Alocodera*, *Apodera*, *Galeripora*, *Bullinularia*, *Cyphoderia*, *Lagenodifflugia*, *Pareuglypha*, and *Quadrulella*. The most abundant species was *Assulinaseminulum*, whereas the least abundant species included *Cyclopyxisarenata* and *Heleopera* (Fig. 3A). Although not among the most abundant, we also documented testate amoeba species typical of southern temperate peatlands, such as *Alocoderacockayni*, *Apoderavas*, *Argynniagertrudeana*, *A.schwabei*, *Certesellacertesi*, *C.martiali*, *Padaungiellawailesi*, *P.wetekampi*, *Sphenoderiaovoidea* and *S.rhombophora* (Fig. 3A). Some of the species recorded are depicted in Fig. 3B–K.

**Figure 4.**
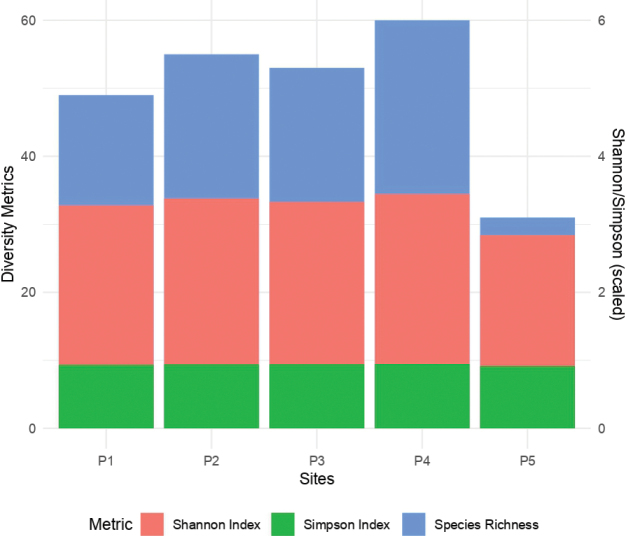
Diversity metrics across study sites P1–P5. Stacked bar plots displaying three diversity metrics: Species Richness (blue), Shannon Index (red), and Simpson Index (green). Each bar represents the cumulative diversity metrics for a given site, allowing comparison of species richness and diversity indices across sites. The secondary y-axis (right) shows scaled values for Shannon and Simpson indices to facilitate interpretation. The variation among sites highlights differences in community structure and evenness.

Species richness, Shannon Index, and Simpson Index were calculated to evaluate the alpha diversity of testate amoebae across five peatlands (P1–P5) (Fig. 4). The results revealed notable variation among the sites. P4 exhibited the highest species richness (60 species), followed by P2 (55), P3 (53), P1 (49), and P5 (31). Similarly, the Shannon Index ranged from 3.45 in P4 to 2.84 in P5, suggesting higher species evenness and diversity in P4 compared to P5. The Simpson Index followed a similar trend, with values ranging from 0.95 in P4 to 0.91 in P5, further supporting the conclusion that P4 harbored the most diverse and evenly distributed communities. These results underscore significant spatial variation in the diversity of testate amoebae among the peatlands.

### ﻿Beta diversity

**Figure 5.**
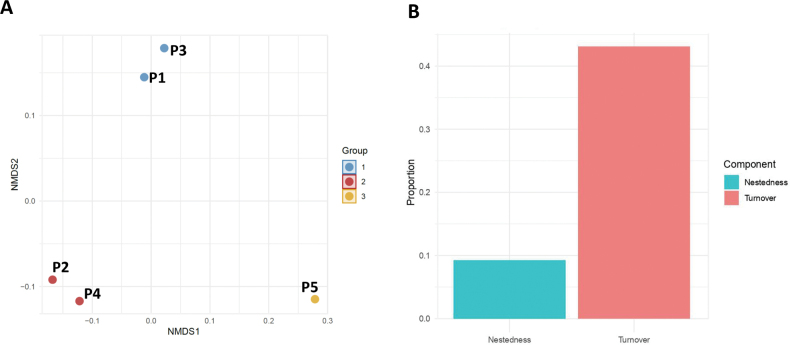
**A** Non-metric multidimensional scaling (nMDS) ordination with k-means clustering. nMDS ordination plot based on Bray-Curtis distance showing the compositional relationships among the sampled sites. The points are colored according to the groups statistically assigned via k-means clustering, highlighting three distinct groups. The visualization highlights spatial differences in species composition between peatlands; **B** partitioning of beta diversity into turnover and nestedness components among the studied peatlands. The bar plot illustrates the proportional contributions of turnover (β_SIM_) and nestedness (β_SNE_) to the total beta diversity (β_SOR_) based on Bray-Curtis dissimilarity. Turnover, accounting for 82.34% of the total beta diversity, suggests that spatial variation in species composition between peatlands is primarily driven by species replacement. In contrast, nestedness contributes only 17.66%, indicating a relatively minor role for species loss or gain. These findings emphasize that these peatland ecosystems harbor distinct communities, as the high turnover reflects substantial species replacement across sites.

The nMDS analysis based on Bray-Curtis similarity suggested a potential grouping pattern among the sampled sites (Fig. 5A). Given the limited number of sites (n = 5), this ordination should be interpreted as an exploratory visualization rather than a definitive classification. The nMDS suggests that site P5 exhibits a distinct composition, whereas sites P1 and P3, as well as sites P2 and P4, appear more similar in species composition.

Betapart analysis, which is less sensitive to sampling site, suggested that beta diversity or variation in species composition (β_SOR_) among the five peatlands was 0.524, with spatial turnover (β_SIM_) accounting for 0.431 and species nestedness (β_SNE_) contributing 0.092. These results indicate that turnover (species replacement) is the primary driver of beta diversity across peatlands, while nestedness (species loss or gain) plays a minor role. The proportional contribution of turnover and nestedness components to beta diversity is visually depicted in Fig. 5B, highlighting the dominance of turnover in shaping community composition across the studied peatlands.

### ﻿Drivers of testate amoeba diversity

Redundancy analysis (RDA) revealed that the first two axes captured a substantial portion of the constrained variation in testate amoeba community structure. RDA1 accounted for 44.59% of the total explained variance, while RDA2 contributed an additional 21.21%, bringing the cumulative variance explained by these two axes to 65.80%. This indicates that the main environmental gradients structuring the community are well represented within the first two dimensions of the ordination space. Organic phosphorus (P-org) exhibited the strongest positive association with RDA1 (0.707), whereas pH showed a strong negative correlation (-0.911), highlighting their dominant roles in shaping the observed species distributions. Sulfate was also an important factor, though its influence was distributed across multiple axes, with a moderate negative association with RDA1 (-0.347) and a positive contribution to RDA2 (0.377). The percentage of explained variance for each identified variable is presented in Table 1.

**Table 1.** Contribution of explanatory variables to testate amoebae community composition. The weights indicate the relative contribution (%) of environmental variables measured at five near-pristine peatlands in the Aysén region, southern Chile, explaining variation in testate amoeba community composition as determined by redundancy analysis (RDA).

Variable	RDA1	RDA2	Interpretation
Sulfate	-0.347	0.377	Negatively contributes to RDA1 but positively to RDA2
Organic phosphorous	0.708	0.381	Strongly and positively contributes to both RDA1 and RDA2
Dissolved organic carbon	0.062	-0.208	Has minimal influence on RDA1 but negatively contributes to RDA2
pH	-0.911	0.408	Negatively contributes to RDA1 and positively to RDA2
Temperature	0.043	-0.012	Exhibits minimal contributions to both RDA1 and RDA2

Variables such as pH and organic phosphorus appear to be the most influential in species variation due to their high coefficients.

Species scores highlighted the differential responses of testate amoebae to these environmental gradients (Suppl. material 1: table S3). For example, *Assulinaseminulum* and *A.muscorum* were positively associated to organic phosphorous along the RDA1, while species like *Centropyxisaerophila* and *Argynniavitraea* showed negative associations. Interestingly, species typical of southern temperate peatlands, such as *Alocoderacockayni*, *Apoderavas*, *Argynniagertrudeana*, *Certesellacertesi*, *C.martiali*, *Sphenoderiaovoidea*, and *S.rhombophora* (Fig. 3), exhibited differential associations with key environmental variables such as pH, organic phosphorus, and sulfate.

**Figure 6.**
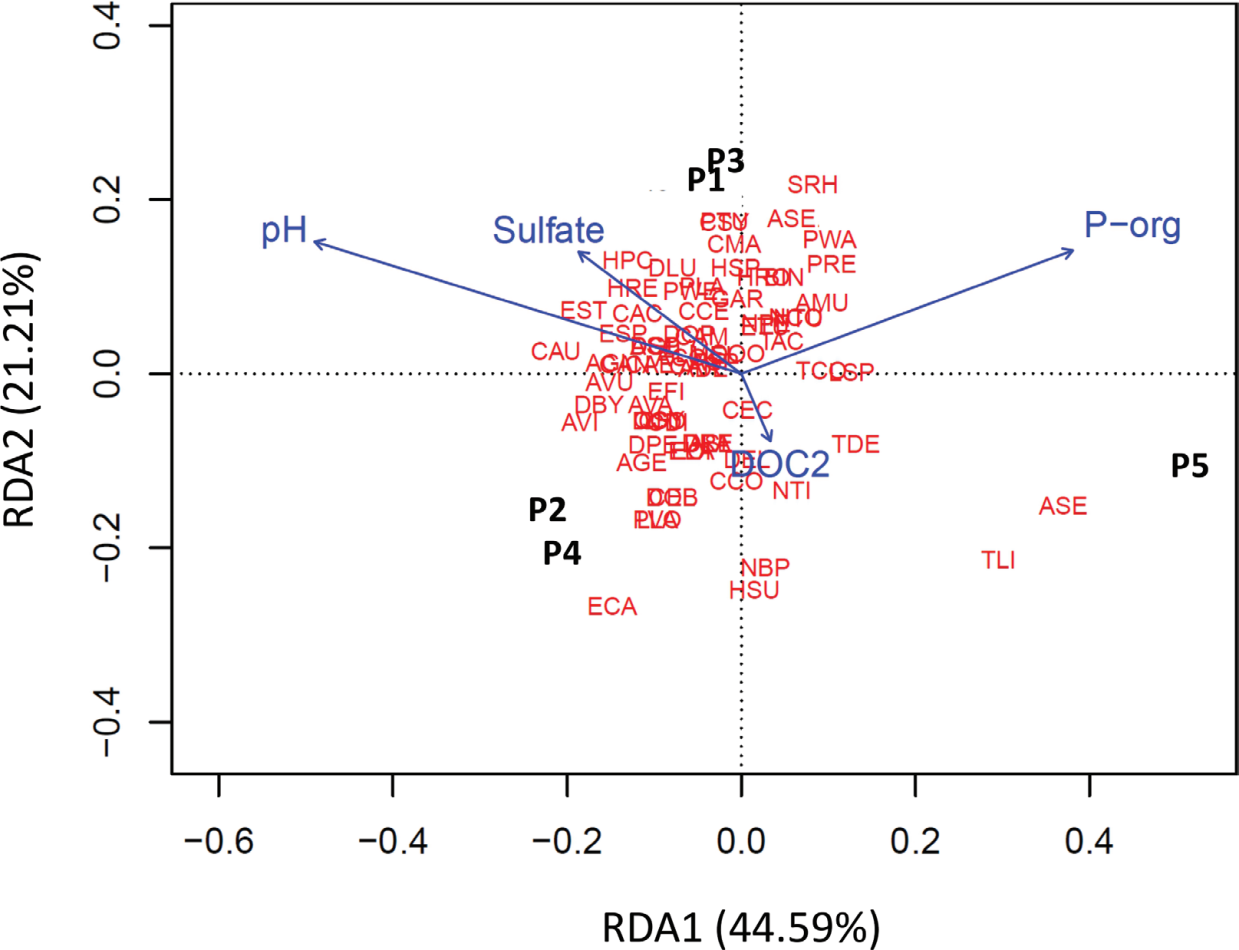
Redundancy Analysis (RDA) biplot showing the relationship between testate amoebae communities and environmental variables across five peatland sites (P1 to P5). The RDA1 axis explains 44.59% of the variation in the dataset, while the RDA2 axis accounts for an additional 21.21%. Environmental variables (blue vectors) represent the environmental gradients that influence the distribution of species (red letters). pH and sulfate showed strong negative associations with RDA1, while organic phosphorus (P-org) had a strong positive association with this axis. The species codes used in this figure correspond to those presented in Fig. 3, where the full list of species and their respective abbreviations are provided for reference.

The site scores reflected clear spatial structuring of the community across the five peatlands (P1 to P5), with higher sulfate concentrations associated with sites P1 and P3, while sites P4 and P5 showed stronger associations with organic phosphorus (Fig. 6).

These findings underscored the importance of specific abiotic factors, particularly organic phosphorous, sulfate, and pH, in shaping microbial communities in peatland ecosystems. Detailed species responses and statistical outputs are provided in Table 1 and in the Suppl. material 1: table S3 for further reference.

## ﻿Discussion

The study of protist diversity and ecology in Chile boats a long tradition (e.g., Certes 1889; Izquierdo 1906; Jung 1942), yet advancements in this field have been limited (Fernández et al. 2015; Campello-Nunes et al. 2022). This knowledge gap is largely due to the Linnean shortfall, characterized by the limited number of taxonomic experts dedicated to protist research, coupled with the considerable challenges involved in sampling and isolating these microorganisms (Fernández et al. 2015; Geisen et al. 2018; Campello-Nunes et al. 2022). These difficulties are further exacerbated by the remoteness of many ecosystems in southern Chile and the lack of conventional access routes to these environments (Fernández 2010; Parra-Gómez and Fernández 2022; Pérez-Schultheiss et al. 2024; Porta et al. 2024; Fernández and Marchant 2025). Testate amoebae are no exception to this trend, and our understanding of their diversity and ecology in Chile remains rudimentary (Fernández et al. 2015). This study addresses these gaps by providing several key contributions: it represents one of the first attempts to document the diversity and ecological drivers of testate amoebae in the undisturbed peatlands of the Chilean Patagonia; it highlights the environmental factors shaping their community composition; and it underscores the importance of preserving these remote ecosystems as critical reservoirs of microbial biodiversity.

### ﻿Alpha diversity

The peatlands of the Aysén region, Chile, revealed a total of 73 testate amoeba species, a remarkably high number compared to previous studies in Patagonian peatlands. For instance, Fernández et al. (2015) reported 110 species across all Chilean temperate peatlands, while Burdman et al. (2021) documented 119 species across temperate Argentinean peatlands and some lakes. The high diversity observed in Aysén may be linked to the pristine condition of these peatlands, which remain virtually undisturbed due to their remote and inaccessible locations. McKeown et al. (2024) reported similar findings in New Zealand, where pristine peatlands harbored a higher diversity of Southern Hemisphere testate amoebae compared to disturbed sites. These findings suggest that the Aysén peatlands serve as a significant reservoir of microbial diversity in Chile, potentially harboring rare or previously unrecorded microbial taxa. Furthermore, recent evidence suggests that climate warming could result in significant range contractions and local extinctions for some Southern Hemisphere testate amoeba species, highlighting the vulnerability of these microbial communities to climate change and further underscoring the importance of conserving these pristine habitats (Bruni et al. 2024).

Furthermore, the actual diversity of testate amoebae in Chilean peatlands, particularly in Aysén, might be underestimated. These protists exhibit considerable morphological variability; for example, *Apoderavas*, a common and emblematic species of Patagonian peatlands, comprises at least nine distinct morphotypes (Zapata and Fernández 2008). Morphological distinctions in some taxa may indicate pseudocryptic species complexes. Genetic evidence supports this, showing that morphologically distinct forms, previously lumped into a single taxon, are indeed separate pseudocryptic species with distinct ecological preferences (Singer et al. 2018). The Patagonian region is increasingly recognized as a microbial diversity hotspot (Fernández et al. 2016; Schiaffino et al. 2016), frequently yielding new protist taxa (Lara et al. 2017) and revealing high genetic diversity within and among protist populations (Fernández et al. 2017). Thus, future molecular approaches are likely to unveil an even greater diversity of testate amoebae in the Aysén peatlands than reported in this study.

### ﻿Beta diversity

The beta diversity analyses highlighted spatial turnover (species replacement) as the dominant phenomenon driving community differentiation among the sampled peatlands. This indicates that species composition varies significantly between peatlands, suggesting that each peatland harbors a unique community of testate amoebae despite sharing similar geographical and climatic characteristics. Such distinct community assemblages underscore the importance of local environmental factors in shaping microbial diversity at a fine spatial scale. In terms of conservation, these findings emphasize the need to protect Patagonian peatlands as discrete ecological units, recognizing that the loss of any single peatland could mean the irreversible loss of unique microbial lineages and the ecosystem functions they support (Fernández 2021; León et al. 2021).

While the nMDS provided an informative visualization of the community patterns, the limited number of sampling sites (*n* = 5) imposes constraints on the robustness of these results. Ordination techniques like nMDS typically require larger datasets to reliably capture gradients in species composition, as small sample sizes may lead to reduced statistical power. Nevertheless, despite this limitation, the clear differentiation observed in the ordination plot suggests that the structure of testate amoeba communities is influenced by underlying environmental variables rather than random variation. Similarly, while beta diversity partitioning (betapart) is generally more resilient to sample size limitations, future studies covering a broader spatial scale could refine our understanding of the relative contributions of species turnover and nestedness to community variation. Despite these limitations, these analyses indicate that beta diversity patterns reflect substantial variation in species composition among peatlands, highlighting that each peatland harbors a distinct community, even when sites are separated by only a few kilometers.

Spatial turnover (i.e., species replacement among peatlands) appears to be the main phenomena underlying beta diversity in testate amoebae across large spatial scales. Turnover explicitly shapes the latitudinal variation in species composition between biomes (Fernández et al. 2016) and implicitly influences genetic variation within the Holarctic region (Singer et al. 2019) as well as species distribution patterns between continents and islands (Fournier et al. 2016) and along environmental gradients (Fernández et al. 2022). These findings suggest that turnover plays a crucial role in structuring testate amoebae communities not only at local levels but also across broad spatial scales. Recognizing this pattern highlights the need for broader conservation strategies that account for the unique microbial diversity present in different biogeographical contexts, particularly in less-studied ecosystems such as Patagonian peatlands.

### ﻿Drivers of testate amoeba diversity

Organic phosphorus, pH, and sulfate emerged as the primary environmental factors shaping the community structure of testate amoebae, with their strongest influence observed along the RDA1 axis. Organic phosphorus displayed a clear positive relationship with this axis, while pH showed a negative association.

Phosphate levels in our studied peatlands ranged from 0.49 to 1.25 µg/l, with an average of 0.83 µg/l, significantly lower than the 10 to 260 µg/l reported in anthropized peatlands (Carballeira and Pontevedra-Pombal 2021). This confirmed that our sites remain largely unaffected by anthropogenic interventions, as the low phosphate concentrations suggest minimal external nutrient inputs. Phosphate and other nutrient-related variables are known to regulate the abundance of testate amoebae by shaping the availability of their microbial food sources (Mieczan and Adamczuk 2015). This relationship is reflected in our RDA results, which indicate that organic phosphorus is positively associated with RDA1, explaining a significant portion of the variation in community composition.

Our findings reveal distinct patterns in the response of different testate amoeba taxa to phosphate levels. Generalist species such as *Difflugiabryophila*, *D.penardi*, and *Cyclopyxisarcelloides* showed stronger associations with sites characterized by higher phosphate concentrations, suggesting that these taxa thrive under nutrient-rich conditions. In contrast, Southern Hemisphere species such as *Alocoderacockayni*, *Apoderavas*, *Certesellacertesi*, and *C.martiali* were more abundant in sites with lower phosphate concentrations. These species are predominantly associated with *Sphagnum*-dominated peatlands, which are naturally nutrient-poor environments (Fernández et al. 2015). Their sensitivity to elevated phosphate levels suggests that they may serve as valuable bioindicators of anthropogenic nutrient contamination in these ecosystems.

Peatlands showed pH values ranging from acidic to slightly acidic, with a minimum of 4.8 and a maximum of 6.3, which are typical for *Sphagnum*-dominated ecosystems worldwide (Parish et al. 2008; Domínguez and Vega 2015; Bonn et al. 2016; Domínguez and Martínez 2021). The strong influence of pH on testate amoeba communities observed is consistent with previous findings, where pH has often been identified as a key factor controlling species distribution in peatlands (Mitchell et al. 1999; Booth 2001; Lamentowicz et al. 2007; Fernández and Zapata 2011; Singer et al. 2018; Carballeira and Pontevedra-Pombal 2021). Species like *Assulinamuscorum* and *Centropyxisaerophila* are known to tolerate a wide range of pH levels (Lamentowicz et al. 2007) and were among the most frequently observed across the studied sites. In contrast, other species, such as *Difflugiaglobulosa* and *Cyclopyxisarcelloides*, showed preferences for slightly more acidic conditions, a pattern consistent with observations from previous studies in peatlands (Mitchell et al. 1999; Booth 2001).

Our study also highlights the presence of several less-studied species typical of Southern Hemisphere peatlands, such as *Al.cockayniA.vas*, *Ar.gertrudeanaC.certesi*, and *C.martiali*. These taxa, which are absent from most studies conducted in Northern Hemisphere peatlands (Fournier et al. 2016; Küppers et al. 2020), showed varying responses to pH gradients. For example, *A.vas* was frequently found in peatlands with slightly acidic pH, while *C.certesi* and *C.martiali* were more abundant in sites with lower pH values. *Ar.gertrudeana* and *Al.cockayni* also appeared to exhibit preferences for specific pH conditions, although their ecological tolerances remain largely unknown. These findings emphasize the need for further ecological research on these unique taxa, particularly in understudied peatland ecosystems of the Southern Hemisphere.

Comparisons with other studies in the Southern Hemisphere suggest that testate amoebae characteristic of these peatlands may serve as key indicators of pristine environmental conditions. McKeown et al. (2024) reported that in New Zealand peatlands, species such as *A.vas* were more abundant in undisturbed sites, while their presence declined significantly in degraded areas. This pattern aligns with our findings in Aysén, where *A.vas* was frequently observed in sites with low sulfate concentrations and moderate pH levels, reinforcing its potential as a bioindicator of natural conditions. Similarly, Bamforth (2015) documented compositional differences in testate amoeba communities across the temperate rainforests of New Zealand and Tasmania, emphasizing the role of hydrological regimes in structuring microbial assemblages.

In Patagonian peatlands, Van Bellen et al. (2014) used testate amoebae as proxies for Holocene hydrological reconstructions, demonstrating that these communities effectively reflect historical changes in water table depth. This approach is relevant to our findings in Aysén, where moisture gradients and pH appear to shape the distribution of key taxa such as *C.certesi* and *C.martiali*. Likewise, Zheng et al. (2019) applied testate amoebae as hydrological proxies in southeastern Australia, showing that they can be powerful indicators of past water table fluctuations. Our results complement these studies by expanding the ecological knowledge of testate amoebae in Southern Hemisphere peatlands and highlighting their relevance for both contemporary monitoring and palaeoecological reconstructions.

Collectively, these comparisons reinforce the idea that testate amoeba communities in the Southern Hemisphere exhibit distributional patterns driven by local environmental factors and hydrological gradients. The consistency between our observations in Aysén and findings from New Zealand, Tasmania, Argentine Patagonia, and Australia further supports the importance of testate amoebae as bioindicators in peatland ecosystems. Moreover, given that Southern Hemisphere testate amoebae remain understudied compared to their Northern Hemisphere counterparts, our results underscore the need for continued research on their ecology and responses to environmental change across different regions of the global south.

An interesting aspect of our findings is the distinct testate amoeba assemblage observed at P5, which differs notably from the communities found in P1–P4. This may be attributed to its unique geographical context, as P5 is located in a different landscape setting compared to the other sites. Unlike P1–P4, which are in close proximity to each other and primarily dominated by *Sphagnum* mosses, P5 is a mixed peatland with a greater presence of vascular plants. Such structural differences in vegetation and hydrology could influence testate amoeba diversity by altering habitat availability, moisture retention, and nutrient cycling. Further research incorporating hydrological measurements and microhabitat characterization would help elucidate the mechanisms underlying these differences.

While the concentrations of pH and organic phosphorus were the most significant predictors along the RDA1 axis, sulfate also showed a strong association with specific sites, particularly P1 and P3, which exhibited higher sulfate concentrations compared to the other peatlands. The observed sulfate concentrations (ranging from 25.61 to 30.64 µg/L) fall within the naturally occurring range for peatlands (Blodau et al. 2007; Schmalenberger et al. 2007) and are likely linked to natural sources rather than anthropogenic pollution. In contrast, much higher sulfate levels have been reported in anthropized peatlands (Carballeira and Pontevedra-Pombal 2021), highlighting the impact of human activities in altering nutrient dynamics in these ecosystems.

Given the proximity of active volcanoes in the region, such as the Hudson Volcano (Fig. 1), the most active in the Chilean Patagonia, it is plausible that volcanic activity contributed to the sulfate levels observed in the studied peatlands. The Hudson Volcano had a major eruption in 2011, during which westerly winds dispersed volcanic tephra eastward toward the sampling sites (Amigo et al. 2012), potentially depositing sulfate into these ecosystems. High sulfate levels may chemically stress testate amoeba communities by altering the ionic balance of water and reducing pH, potentially creating unfavorable conditions for certain taxa (Lamentowicz et al. 2007). This aligns with Payne et al. (2010), who reported decreased species richness in peatlands with high sulfate levels, attributing this pattern to changes in the bacterial composition driven by sulfate. Such shifts in the microbial community may alter the availability of food resources, as some testate amoebae exhibit feeding preferences for specific bacterial taxa. Interestingly, sites P1 and P3, which were strongly associated with sulfate in the RDA, also exhibited comparatively lower species richness than the peatlands with moderate or low sulfate concentrations (Fig. 4). Notably, volcanic eruptions may also influence testate amoeba community structure by promoting the dominance of agglutinated taxa, which construct their shells by incorporating surrounding mineral particles. These taxa may benefit from the influx of cryptotephra as an abundant and suitable building material (Delaine et al. 2016), potentially shaping assemblages following major volcanic events.

Our redundancy analysis (RDA) revealed clear associations between environmental variables and testate amoeba community structure. However, we were unable to formally assess the statistical significance of the model due to the limited number of sampling sites (n = 5). Permutation-based ANOVA tests require an adequate number of replicates to estimate residual variance and generate meaningful significance values. In our case, the model explained all the variability (R^2^ = 1), but the lack of residual degrees of freedom prevented the calculation of valid F or *P*-values. This limitation highlights the need for a larger sample size in future studies to enable more robust statistical validation of constrained ordination models.

Similar to Lamentowicz et al. (2007), our study also found that sites with ‘high’ sulfate levels contained abundant agglutinated species, such as *Difflugiabryophila*, *D.penardi*, and *Cyclopyxisarcelloides*. Volcanic tephra, rich in silicate particles, provides a crucial physical resource for these species to construct their shells. Delaine et al. (2016) demonstrated that tephra particles can be readily incorporated into the shells of agglutinated testate amoebae after volcanic eruptions. Interestingly, these are the same species that were also abundant in peatlands with elevated phosphate levels, indicating that they exhibit generalist ecological strategies in temperate peatlands of Aysén. In our study, this suggests that both physical factors, such as the availability of silicate particles from volcanic tephra, and chemical factors, such as sulfate concentrations, may play complementary roles in shaping the distribution of agglutinated species across different peatland sites.

These findings suggest a dual influence of volcanic activity on testate amoeba communities: sulfate acts as an environmental filter, potentially reducing species richness by promoting the dominance of generalist species, whereas tephra serves as a physical resource that supports the proliferation of agglutinated species. This interaction underscores the complex interplay between natural abiotic factors and the structuring of microbial communities in southern peatlands.

Our results also highlight an interesting contrast in species richness and diversity between P4 and P5. P4 exhibited the highest species richness among all sites, while P5 had the lowest diversity. This pattern may be linked to differences in environmental conditions. P4 was characterized by moderate pH levels and relatively balanced nutrient availability, which may provide a favorable environment for a diverse community of testate amoebae. In contrast, P5 had higher concentrations of sulfate and lower pH, conditions that could act as environmental filters favoring a subset of tolerant taxa while excluding others. These findings align with previous studies showing that high sulfate concentrations and lower pH levels can reduce testate amoeba diversity by altering microbial food web interactions and habitat suitability (Lamentowicz et al. 2007; Payne et al. 2010). Future studies should aim to disentangle these potential effects by examining additional physicochemical parameters, microbial interactions, and microtopographical heterogeneity within these peatlands.

## ﻿Conclusions

The findings of this study contribute to a growing body of evidence highlighting the unique ecological dynamics of southern Chilean peatlands. The high turnover observed suggests that each peatland harbors distinct testate amoeba communities, emphasizing their conservation value. In a global context, these results reinforce the role of peatlands as reservoirs of microbial diversity, particularly in underexplored regions like the Chilean Patagonia.

Comparing the results from Aysén to studies in other regions underscores the biogeographic variability in testate amoeba community dynamics and environmental drivers. These insights highlight the need for region-specific conservation strategies that account for the unique ecological characteristics of peatlands. The near-pristine nature of Aysén’s peatlands offers a valuable reference for understanding the natural dynamics of these ecosystems and the potential impacts of climate change and anthropogenic disturbances.

Future research should expand on these findings by exploring the functional roles of testate amoebae in peatland ecosystems and their interactions with other microbial (bacteria, protist, fungal) communities. Additionally, integrating molecular approaches with traditional morphological methods could provide deeper insights into the biodiversity and ecological functions of these protists in peatlands globally.

## ﻿Additional information

### Author ORCIDs

Leonardo D. Fernández https://orcid.org/0000-0001-9550-1921

Erwin Domínguez https://orcid.org/0000-0002-1907-8399

Antonio Parra-Gómez https://orcid.org/0000-0002-4144-6610

Enrique Lara https://orcid.org/0000-0001-8500-522X

### Data availability

All of the data that support the findings of this study are available in the main text or Supplementary Information.

## Supplementary materials

Supplementary material 1 Supplementary tables and figure Data type docx Explanation note **table S1.** Water samples were collected from peatlands (P1–P5) following standard protocols for environmental monitoring; **table S2.** Relative abundance of testate amoebae taxa in each peatland studied (P1 to P5); **table S3.** Testate amoeba species scores from redundancy analysis (RDA); **figure S1.** Correlation analysis of environmental variables. https://binary.pensoft.net/file/1334979This dataset is made available under the Open Database License (http://opendatacommons.org/licenses/odbl/1.0/). The Open Database License (ODbL) is a license agreement intended to allow users to freely share, modify, and use this Dataset while maintaining this same freedom for others, provided that the original source and author(s) are credited.Leonardo D. Fernández, Erwin Domínguez, Antonio Parra-Gómez, Enrique Lara
