# Supplementary material for: ﻿Protist ecology in Patagonian peatlands: pH, organic phosphorus, and sulfate as key drivers of testate amoeba diversity in undisturbed ecosystems
Source: Zookeys. 2025 May 21;1239:75–101. doi: 10.3897/zookeys.1239.146538 (PMC12120495; doi:10.3897/zookeys.1239.146538)
Supplement: Supplementary material 1 — Supplementary tables and figure [file zookeys-1239-075_article-146538__-s001.docx]

**Supplementary material**

**Protist ecology in Patagonian peatlands: pH, organic phosphorus, and sulfate as key drivers of testate amoeba diversity in undisturbed ecosystems**

Leonardo D. Fernández, Erwin Domínguez, Antonio Parra Gómez, Enrique Lara

**Table S1.** Water samples were collected from peatlands (P1-P5) following standard protocols for environmental monitoring. At each sampling site, water was collected in acid-washed polyethylene bottles to minimize contamination. Parameters such as pH and temperature were measured in situ using a portable multiparameter probe. Samples for dissolved ions i.e., dissolved silica, alkalinity, sodium, potassium, magnesium, calcium, fluoride, chloride, and sulfate; as well as nutrient concentrations i.e., ammonium, nitrate-nitrite nitrogen, organic nitrogen, orthophosphate phosphorus, organic phosphorus, were filtered through 0.45 µm membranes in the field and preserved at 4°C or acidified (using H₂SO₄ or HCl) depending on the target analyte. Dissolved organic carbon was measured in the laboratory using UV-Vis spectrophotometry for absorbance (DOC1) and total organic carbon analysis (DOC2). All samples were analyzed in a certified laboratory employing appropriate techniques, including ion chromatography, spectrophotometry, and ICP-OES (Inductively Coupled Plasma Optical Emission Spectrometry) for ion concentrations.

|  | Peatlands (study sites) | | | | |
| --- | --- | --- | --- | --- | --- |
| Parameters | P1 | P2 | P3 | P4 | P5 |
| Dissolved silica (µM/l) | 117.23 | 69.14 | 65.06 | 78.53 | 122.96 |
| Alkalinity (mgCaCO3/l) | 25.84 | 7.74 | 11.81 | 12.09 | 37.57 |
| Sodium (µg/l) | 1744.83 | 1262.64 | 1348.55 | 1287.91 | 1872.49 |
| Ammonium (µg/l) | 24.68 | 94.42 | 102.77 | 36.76 | 122.14 |
| Potassium (µg/l) | 220.80 | 233.63 | 310.35 | 216.47 | 285.07 |
| Magnesium (µg/l) | 482.75 | 251.51 | 278.01 | 295.14 | 652.40 |
| Calcium (µg/l) | 10480.47 | 3214.65 | 7298.20 | 5321.33 | 15705.46 |
| Fluoride (µg/l) | 0.68 | 0.46 | 0.47 | 0.45 | 0.51 |
| Chloride (µg/l) | 263.35 | 146.04 | 143.45 | 148.13 | 170.78 |
| Sulfate (µg/l) | 30.64 | 27.16 | 25.61 | 26.47 | 25.72 |
| Nitrate-nitrite nitrogen (µg/l) | 41.01 | 40.18 | 41.12 | 38.96 | 39.09 |
| Organic nitrogen (µg/l) | 14.08 | 13.66 | 11.58 | 17.33 | 21.08 |
| Orthophosphate phosphorus (µg/l) | 3.95 | 5.58 | 3.57 | 3.42 | 23.67 |
| Organic phosphorus (µg/l) | 1.24 | 0.49 | 0.64 | 0.53 | 1.25 |
| DOC1 (Abs total) | 1583.86 | 2431.47 | 1065.17 | 1049.36 | 1505.67 |
| DOC2 (Pend UV) | 0.01 | 0.01 | 0.01 | 0.01 | 0.01 |
| pH | 6.686 | 6.473 | 6.529 | 6.395 | 5.414 |
| Temp (°C) | 4.9 | 4.2 | 5.3 | 6.3 | 5.9 |

**Table S2.** Relative abundance of testate amoebae taxa in each peatland studied (P1 to P5). The values represent the proportion of individuals from each taxon relative to the total number of individuals found at each site (individuals per gram of dry moss).

| **Taxa** | **P1** | **P2** | **P3** | **P4** | **P5** | **Taxa** | **P1** | **P2** | **P3** | **P4** | **P5** |
| --- | --- | --- | --- | --- | --- | --- | --- | --- | --- | --- | --- |
| ACO | 0.0149 | 0.0243 | 0.051 | 0.0398 | 0.0150 | DRE | 0 | 0 | 0 | 0.0050 | 0 |
| ADE | 0 | 0 | 0.004 | 0.0050 | 0 | DSP | 0.0050 | 0.0049 | 0.0042 | 0.0050 | 0 |
| AGE | 0 | 0.0146 | 0.004 | 0.0050 | 0 | ECA | 0 | 0.0146 | 0 | 0.0199 | 0 |
| AGI | 0.0149 | 0.0097 | 0.004 | 0.0149 | 0 | ECI | 0 | 0.0097 | 0 | 0 | 0 |
| AMU | 0.1733 | 0.0922 | 0.118 | 0.1244 | 0.1654 | EFI | 0 | 0.0049 | 0.0084 | 0.0050 | 0 |
| ASC | 0.0050 | 0.0049 | 0.004 | 0.0050 | 0 | ELA | 0 | 0.0097 | 0 | 0 | 0 |
| ASE1 | 0 | 0 | 0 | 0 | 0.0752 | ESP | 0.0099 | 0.0049 | 0.0084 | 0.0149 | 0 |
| ASE2 | 0.0743 | 0.0437 | 0.0506 | 0.0149 | 0.0602 | EST | 0.0149 | 0.0243 | 0.0169 | 0.0100 | 0 |
| ASI | 0 | 0 | 0 | 0.0050 | 0 | ETU | 0.0099 | 0.0146 | 0.0042 | 0 | 0.0075 |
| ASP | 0.0495 | 0.112 | 0.1392 | 0.0746 | 0.0752 | GAR | 0 | 0 | 0.0042 | 0 | 0 |
| AVA | 0.0248 | 0.049 | 0.0338 | 0.0348 | 0.0150 | HEL | 0.0149 | 0.0194 | 0.0084 | 0.0050 | 0.0075 |
| AVI | 0.1238 | 0.155 | 0.0759 | 0.1144 | 0.0526 | HPC | 0.0149 | 0 | 0.0169 | 0.0299 | 0 |
| AVU | 0.0099 | 0.015 | 0.0042 | 0.0100 | 0 | HRE | 0.0099 | 0.0049 | 0.0127 | 0.0100 | 0 |
| BIN | 0.0248 | 0.019 | 0.0422 | 0.0149 | 0.0301 | HRO | 0.0099 | 0 | 0.0169 | 0.0149 | 0.0075 |
| CAC | 0.0050 | 0.010 | 0.0127 | 0.0050 | 0 | HSP | 0 | 0 | 0.0084 | 0 | 0 |
| CAE | 0.0149 | 0.005 | 0 | 0.0050 | 0 | HSU | 0 | 0.0049 | 0 | 0.0149 | 0.0075 |
| CAM | 0 | 0 | 0.0084 | 0.0050 | 0 | LMO | 0 | 0.0049 | 0.0042 | 0 | 0 |
| CAN | 0.0099 | 0.0049 | 0.0042 | 0.0149 | 0 | LSP | 0 | 0 | 0.0127 | 0.0050 | 0.0150 |
| CAR | 0.0050 | 0 | 0 | 0.0050 | 0 | LVA | 0 | 0.0097 | 0 | 0.0050 | 0 |
| CAU | 0.0149 | 0.0194 | 0.0211 | 0.0299 | 0 | NBP | 0 | 0.0049 | 0 | 0.0100 | 0.0075 |
| CCE | 0.0248 | 0.0097 | 0.0127 | 0.0149 | 0.0075 | NCO | 0.0149 | 0.0049 | 0.0084 | 0.0050 | 0.0150 |
| CCO | 0.0099 | 0.0049 | 0 | 0.0149 | 0.0075 | NPE | 0.0198 | 0.0049 | 0.0127 | 0.0149 | 0.0150 |
| CDI | 0 | 0.0049 | 0.0042 | 0.0050 | 0 | NTI | 0 | 0.0146 | 0.0084 | 0.0050 | 0.0150 |
| CEC | 0.0050 | 0.0097 | 0.0042 | 0.0050 | 0.0075 | NTU | 0.0099 | 0.0049 | 0.0127 | 0.0050 | 0.0150 |
| CEL | 0 | 0.0049 | 0 | 0.0050 | 0 | PLA | 0.0248 | 0.0146 | 0.0169 | 0.0100 | 0.0075 |
| CHI | 0.0050 | 0.0049 | 0 | 0.0050 | 0 | PLO | 0 | 0.0097 | 0 | 0.0050 | 0 |
| CMA | 0 | 0 | 0.0127 | 0 | 0 | PRE | 0.0050 | 0 | 0.0042 | 0 | 0.0075 |
| CSY | 0.0050 | 0 | 0.0042 | 0 | 0 | PTU | 0.0050 | 0 | 0.0042 | 0 | 0 |
| DBY | 0.0099 | 0.0146 | 0.0042 | 0.0149 | 0 | PWA | 0.0149 | 0 | 0.0127 | 0.0050 | 0.0150 |
| DCO | 0.0050 | 0.0049 | 0 | 0.0050 | 0 | PWE | 0.0050 | 0 | 0.0042 | 0.0050 | 0 |
| DEL | 0.0099 | 0.0049 | 0 | 0.0100 | 0.0075 | QSY | 0.0050 | 0.0049 | 0 | 0.0050 | 0 |
| DGL | 0.0050 | 0.0049 | 0.0042 | 0.0050 | 0 | SOO | 0.0149 | 0.0097 | 0.0042 | 0.0050 | 0.0075 |
| DLA | 0 | 0 | 0 | 0.0050 | 0 | SRH | 0.0347 | 0.0194 | 0.0211 | 0 | 0.0226 |
| DLU | 0.0050 | 0.0097 | 0.0084 | 0 | 0 | TAC | 0.0050 | 0.0097 | 0.0042 | 0 | 0.0075 |
| DOB | 0 | 0.0049 | 0 | 0.0050 | 0 | TCO | 0.0149 | 0.0243 | 0.0253 | 0.0100 | 0.0376 |
| DOP | 0.0099 | 0 | 0 | 0.0050 | 0 | TDE | 0.0693 | 0.0583 | 0.0759 | 0.0995 | 0.1353 |
| DPE | 0 | 0.0097 | 0.0042 | 0.0050 | 0 | TLI | 0.0099 | 0.0146 | 0.0211 | 0.0398 | 0.1278 |

ACO: Alocodera cockayni, ADE: Argynnia dentistoma, AGE: Argynnia gertrudeana, AGI: Arcella gibbosa, AMU: Assulina muscorum, ASC: Argynnia schwabei, ASE1: Amphitrema stenostoma, ASE2: *Argynnia seminulum*, ASP: Amphitrema sp., AVA: Apodera vas, AVI: Argynnia vitraea, AVU: Arcella vulgaris, BIN: Bullinularia indica, CAC: Centropyxis aculeata, CAE: Centropyxis aerophila, CAM: Cyphoderia ampulla, CAN: Cyclopyxis arenata, CAR: Cyclopyxis arcelloides, CAU: Certesella australis, CCE: Certesella certesi, CCO: Centropyxis constricta, CEC: Centropyxis ecornis, CEL: Centropyxis elongata, CHI: Centropyxis hirsuta, CMA: Certesella martiali, CSY: Centropyxis sylvatica, DBY: Difflugia bryophila, DCO: Difflugia corona, DEL: Difflugia elegans, DGL: Difflugia globulosa, DLA: Difflugia lacustris, DLU: Difflugia lucida, DOB: Difflugia cf. oblonga, DOP: Difflugia opulenta, DPE: Difflugia penardi, DRE: Difflugia regularis, DSP: Difflugia sp., ECA: Euglypha cristata acicularis, ECI: Euglypha ciliata, EFI: Euglypha filifera, ELA: Euglypha laevis, ESP: Euglypha sp., EST: Euglypha strigosa, ETU: Euglypha tuberculata, GAR: Galeripora arenaria, HEL: Hyalosphenia elegans, HPC: Heleopera cf. petricola, HRE: Heleopera rectangularis, HRO: Heleopera cf. rosea, HSP: Heleopera sp., HSU: Hyalosphenia subflava, LMO: Lesquereusia cf. modesta, LSP: Lesquereusia spiralis, LVA: Lagenodifflugia vas, NBP: Nebela barbata psilonata, NCO: Nebela cf. collaris, NPE: Nebela penardiana, NTI: Nebela cf. tincta, NTU: Nebela tubulosa, PLA: Padaungiella lageniformis, PLO: Padaungiella longitubulata, PRE: Pareuglypha reticulata, PTU: Padaungiella tubulata, PWA: Padaungiella wailesi, PWE: Padaungiella wetekampi, QSY: Quadrulella cf. symmetrica, SOO: Sphenoderia ovoidea, SRH: Sphenoderia rhombophora, TAC: Tracheleuglypha acolla, TCO: Trinema complanatum, TDE: Tracheleuglypha dentata, TLI: Trinema lineare.


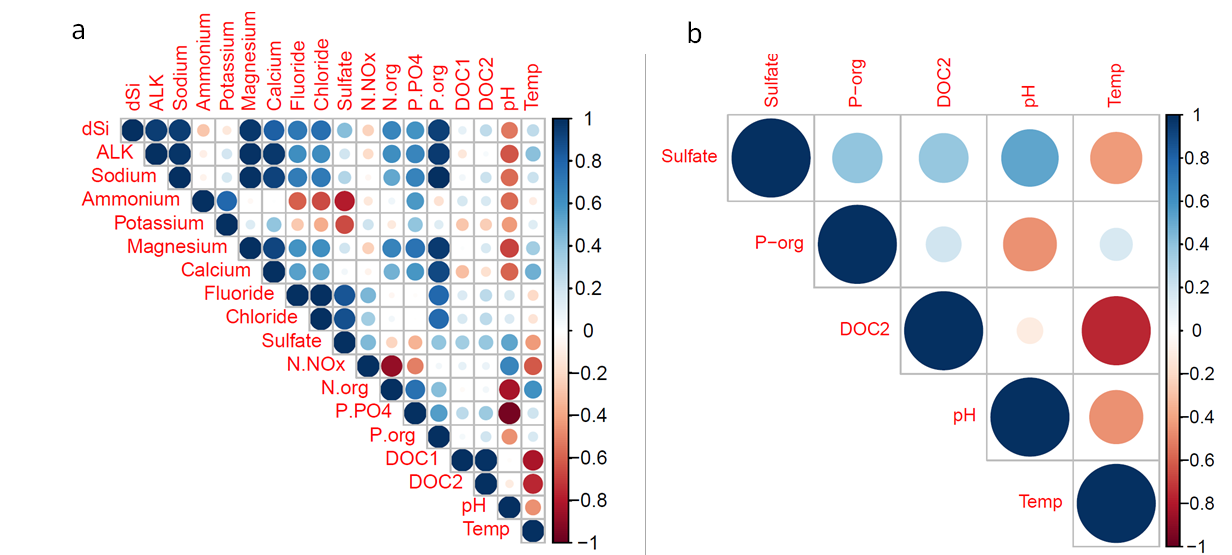


**Figure S1: Correlation analysis of environmental variables.** (a) Pairwise Pearson correlation coefficients among all measured environmental variables visualized as a heatmap. High correlations (r > 0.8) are indicated by larger and darker circles, while weaker correlations are represented by smaller and lighter circles. Variables with high correlations were excluded from further analysis to reduce multicollinearity. (b) Pairwise Pearson correlation coefficients for the subset of retained environmental variables (temperature, pH, dissolved organic carbon, organic phosphorus, and sulfate). The absence of high correlations (r > 0.8) among these variables was confirmed, ensuring their suitability for redundancy analysis (RDA). Circle sizes and colors indicate the strength and direction of correlations, with blue representing positive correlations and red representing negative correlations.

**Table S3. Testate amoeba species scores from redundancy analysis (RDA).** This table presents the species scores along the first four RDA axes, representing the contributions of each species to the variation explained by the environmental gradients. Positive or negative values indicate the strength and direction of association of each species with the respective RDA axes. These scores provide insight into the ecological preferences and responses of the species to specific environmental variables, highlighting key patterns of species-environment relationships in peatland ecosystems. Please refer to Table S2 for the full names of the species corresponding to the codes used in this table.

| Taxa | RDA1 | RDA2 | RDA3 | RDA4 | Taxa | RDA1 | RDA2 | RDA3 | RDA4 |
| --- | --- | --- | --- | --- | --- | --- | --- | --- | --- |
| ACO | -0.046 | 0.008 | -0.019 | 0.088 | DPE | -0.068 | -0.038 | 0.033 | 0.050 |
| ASE | 0.260 | -0.070 | 0.006 | -0.015 | DRE | -0.026 | -0.037 | -0.052 | 0.012 |
| ASP | -0.021 | 0.006 | 0.068 | 0.108 | DSP | -0.066 | 0.015 | -0.003 | 0.000 |
| AVA | -0.070 | -0.016 | 0.031 | 0.020 | ECI | -0.038 | -0.041 | 0.078 | -0.012 |
| GAR | -0.003 | 0.040 | 0.007 | 0.050 | ECA | -0.099 | -0.123 | -0.007 | 0.010 |
| AGI | -0.104 | 0.006 | -0.027 | -0.034 | EFI | -0.058 | -0.009 | 0.013 | 0.074 |
| AVU | -0.101 | -0.005 | 0.011 | -0.023 | ELA | -0.038 | -0.041 | 0.078 | -0.012 |
| ADE | -0.029 | 0.003 | -0.045 | 0.062 | ESP | -0.091 | 0.021 | -0.043 | 0.007 |
| AGE | -0.076 | -0.047 | 0.051 | 0.047 | EST | -0.121 | 0.033 | 0.041 | 0.005 |
| ASI | -0.026 | -0.037 | -0.052 | 0.012 | ETU | 0.018 | 0.025 | 0.086 | -0.045 |
| ASC | -0.066 | 0.015 | -0.003 | 0.000 | HPC | -0.087 | 0.060 | -0.136 | 0.036 |
| AVI | -0.124 | -0.026 | 0.033 | -0.059 | HRE | -0.083 | 0.046 | -0.025 | 0.019 |
| AMU | 0.062 | 0.038 | -0.051 | -0.052 | HRO | 0.017 | 0.051 | -0.093 | 0.040 |
| ASE | 0.057 | 0.082 | 0.054 | -0.053 | HSP | -0.005 | 0.056 | 0.009 | 0.070 |
| BIN | 0.033 | 0.051 | 0.017 | 0.032 | HSU | 0.010 | -0.114 | -0.032 | 0.008 |
| CAC | -0.080 | 0.032 | 0.025 | 0.033 | HEL | -0.020 | 0.010 | 0.048 | -0.032 |
| CAE | -0.070 | 0.005 | -0.019 | -0.089 | LVA | -0.064 | -0.077 | 0.027 | 0.000 |
| CCO | -0.004 | -0.057 | -0.051 | -0.068 | LMO | -0.030 | 0.011 | 0.062 | 0.041 |
| CDI | -0.056 | -0.026 | 0.010 | 0.053 | LSP | 0.085 | 0.001 | -0.038 | 0.091 |
| CEC | 0.005 | -0.019 | 0.022 | -0.008 | NBP | 0.018 | -0.103 | -0.016 | 0.004 |
| CEL | -0.053 | -0.065 | 0.004 | 0.004 | NCO | 0.041 | 0.030 | -0.007 | -0.026 |
| CHI | -0.063 | -0.025 | -0.009 | -0.050 | NPE | 0.019 | 0.026 | -0.046 | -0.015 |
| CSY | -0.013 | 0.080 | -0.006 | -0.004 | NTI | 0.039 | -0.062 | 0.056 | 0.061 |
| CAU | -0.142 | 0.012 | -0.023 | 0.031 | NTU | 0.044 | 0.029 | -0.001 | 0.007 |
| CCE | -0.029 | 0.033 | -0.027 | -0.029 | PLA | -0.030 | 0.046 | 0.009 | -0.023 |
| CMA | -0.006 | 0.069 | 0.011 | 0.086 | PTU | -0.013 | 0.080 | -0.006 | -0.004 |
| CAR | -0.036 | 0.004 | -0.065 | -0.042 | PLO | -0.064 | -0.077 | 0.027 | 0.000 |
| CAN | -0.089 | 0.005 | -0.046 | -0.014 | PWA | 0.068 | 0.071 | -0.060 | -0.002 |
| CAM | -0.031 | 0.019 | -0.042 | 0.082 | PWE | -0.039 | 0.044 | -0.058 | 0.008 |
| DBY | -0.109 | -0.016 | -0.005 | -0.020 | PRE | 0.069 | 0.058 | -0.005 | -0.009 |
| DCO | -0.063 | -0.025 | -0.009 | -0.050 | QSY | -0.063 | -0.025 | -0.009 | -0.050 |
| DEL | 0.004 | -0.045 | -0.034 | -0.072 | SOO | -0.002 | 0.010 | 0.012 | -0.048 |
| DGL | -0.066 | 0.015 | -0.003 | 0.000 | SRH | 0.055 | 0.100 | 0.094 | -0.056 |
| DLA | -0.026 | -0.037 | -0.052 | 0.012 | TAC | 0.031 | 0.017 | 0.074 | -0.020 |
| DLU | -0.053 | 0.056 | 0.074 | 0.005 | TDE | 0.088 | -0.037 | -0.052 | 0.015 |
| DOB | -0.053 | -0.065 | 0.004 | 0.004 | TCO | 0.061 | 0.002 | 0.048 | 0.017 |
| DOP | -0.040 | 0.021 | -0.070 | -0.064 | TLI | 0.197 | -0.098 | -0.046 | 0.036 |
